# Effects of Irrigation Rate on Soil Bacterial Diversity and Lint Yield in a Jujube/Cotton Intercropping System

**DOI:** 10.3390/microorganisms14071413

**Published:** 2026-06-27

**Authors:** Yanfang Li, Yongqi Mu, Guodong Chen, Xu Chen, Wei Zhang, Hong Zeng, Sumei Wan

**Affiliations:** 1 College of Agriculture, Key Laboratory of Genetic Improvement and Efficient Production for Specialty Crops in Arid Southern Xinjiang of Xinjiang Corps, Tarim University, Alar 843300, China; 107572021304@stumail.taru.edu.cn (Y.L.); guodongchen@taru.edu.cn (G.C.); chenxu19921019@163.com (X.C.); 2 College of Life Science and Technology, Key Laboratory of Protection and Utilization of Biological Resources in Tarim Basin of Xinjiang Production & Construction Corps, Tarim University, Alar 843300, China; muyq1205@163.com; 3 College of Agriculture, Shihezi University, Shihezi 832003, China; bluesky2002040@shzu.edu.cn; 4 School of Basic Medicine, Youjiang Medical University for Nationalities, Baise 533000, China

**Keywords:** jujube/cotton intercropping, irrigation rate, enzymatic activities, microbial diversity, lint yield

## Abstract

Jujube/cotton intercropping is a typical ecological ecosystem, and water is the dominant factor limiting the system productivity. However, it is not clear if and how irrigation rate affects soil bacterial diversity and lint yield in a jujube/cotton intercropping system. To explore the effects of irrigation rate on soil bacterial diversity and enzymatic activities in a jujube/cotton intercropping system, four water stress treatments were applied in 2017 and 2018 in Xinjiang, Northwest China: (1) W1, 3750 m^3^/ha; (2) W2, 4500 m^3^/ha; (3) W3, 5250 m^3^/ha; (4) W4, 6000 m^3^/ha. In the intercropping field, cotton yield in W2 and W3 treatment was higher than that in W1 and W4 treatments, and significant increases were also observed in soil available phosphorus and potassium, as well as soil alkaline phosphatase and urease activities. The richness and diversity of soil bacterial communities in W2 and W3 treatments were higher than in other treatments, and the abundance of *Pseudomonas* bacteria was highest under W2 and W3 treatments. Overall, soil nutrient levels and soil enzymatic activities influenced soil bacterial community structure in intercropping system, indicating that soil environment was effectively improved and the best farmland income was obtained when the irrigation rate was controlled at 4500 to 5250 m^3^/ha.

## 1. Introduction

Xinjiang is a major cotton-producing region, where the rainfall during the growing season is low, high rates of transpiration and evaporation, and high summer temperatures limit the availability of soil moisture [1]. Recent trends in precipitation and temperature have negatively impacted cotton yields in the arid upper southern region of Xinjiang. Some crops exhibit heightened sensitivity to water deficits, which can result in substantial yield reductions even in the absence of visible water stress indicators [2]. Southern Xinjiang, China, has a temperate continental dry climate with low rainfall, high temperatures, long sunshine hours, and high evapotranspiration, making drought a key factor limiting crop productivity in the region [3].

Previous studies have demonstrated that water stress significantly affects soil chemical properties, enzymatic activities, and microbial communities. Water deficit has been shown to alter soil extracellular enzyme activities, with severe drought significantly increasing soil urease activity while decreasing bacterial richness and shifting bacterial community structure [4]. Drought conditions also reduce the maximum reaction rates of C-, N-, and P-degrading enzymes, particularly in drier ecosystems [5]. At the community level, soil moisture content is positively correlated with microbial abundance, enzyme activity, and functional diversity, with drought-tolerant taxa such as *Firmicutes* and *Gemmatimonadota* exhibiting resistance under water-limited conditions [6]. Under extreme drought, bacterial communities in rhizosphere soil show enriched *Firmicutes* and *Proteobacteria*, and drought-resistant crops develop more complex microbial co-occurrence networks [7]. However, most of these studies have been conducted in monoculture systems or natural grasslands, leaving a knowledge gap regarding how water stress influences soil properties and microbial communities in intercropping systems.

Cotton is a major crop in Xinjiang, and jujube/cotton agroforestry systems play an important role in southern Xinjiang. Under limited water availability, intercropping enhances water use efficiency [8]. Farmers strive to maximize the use of limited land, water, sunlight, and heat resources to increase economic yield while enhancing the sustainable development and stability of agroforestry systems. Additionally, jujube/cotton agroforestry systems can alter the farmland microclimate [9]. Soil microbial and enzymatic activities are crucial components of soil ecosystems, enhancing soil fertility and ecosystem health [10].

Over the past few decades, jujube/cotton agroforestry systems have improved re-source utilization efficiency [11]. Intercropping can improve soil nutrients, biodiversity, and enzymatic activity [12]. In response to water stress, crops grow more roots to access additional soil resources [13]. Dry soil creates suboptimal conditions for soil microbial life, leading to fluctuations in soil metabolic activity. Soil nutrient availability is mainly determined by soil moisture content, as most plant nutrients are transported to plant roots through mass flow [13]. In intercropping system, crop roots intertwine, enhancing root exudates that support a variety of soil microorganisms [14]. An optimal soil environment can increase microbial populations and enzymatic activity, promote crop shoot growth, and boost crop yield [15].

Despite the recognized importance of water stress on soil properties, most research in jujube/cotton intercropping systems has focused on yield and growth characteristics rather than soil microbial and enzymatic responses [16,17]. Consequently, limited information exists on the effects of water stress on soil bacterial diversity and enzymatic activity in jujube/cotton intercropping systems, and how soil moisture influences the spatial distribution of microbial diversity remains unclear.

Therefore, this study was conducted in a jujube/cotton system in the Alar region of the Tarim Basin (Southern Xinjiang) to address the following objectives: (1) evaluate soil chemical properties and crop yield under different water stress levels in intercropped cotton fields, (2) identify irrigation treatments that influence soil bacterial community structure and enzymatic activity, and (3) analyze how water stress affects soil bacterial community changes to improve soil conditions in intercropping system and provide theoretical guidance for irrigation practices in intercropping systems in arid regions of Southern Xinjiang.

## 2. Materials and Methods

### 2.1. Experimental Site and Soil Conditions

This study was conducted at Tarim University (40°32′ N, 81°18′ E) in the Eastern district of Alar, Xinjiang, Northwest China. Alar is characterized by a temperate continental dry climate. The average annual temperature is 10.8 °C, annual precipitation is 40.10–82.50 mm, and annual evaporation of 1976.6–2558.9 mm. The area basks in sunshine 2996 h per year. The soil is sandy loam with pH 7.90; available phosphorus (AP) is 58.70 mg/kg soil; available potassium (AK) is 107.34 mg/kg soil; total nitrogen (TN) is 1.51 mg/kg soil; and organic matter (OM) is 11.20 mg/kg soil.

The intercrop study was a randomized complete block design with a 1:4 intercrop ratio (one row of jujube and four rows of cotton). The crop system was replicated three times. The plot size was 10 m × 3 m with a 1.0 m distance between the crop rows in the intercrop system. The experimental intercrop system was established in 2012, and the soil used in the present study was collected during 2017 and 2018. For cotton, Xinluzhong-38 (*Gossypium hirsutum* L.), a dominant cotton cultivar in the local area, was used for the experiments. For jujube, the Huizao cultivar (*Ziziphus jujuba* Mill.) was used.

### 2.2. Irrigation Treatments

The irrigation amounts in this study were designed based on previous research on mulched drip irrigation for cotton in southern Xinjiang [18]. According to these studies, maintaining soil water content at 75–85% of field capacity (FC) after irrigation is considered conventional field irrigation practice, representing the optimal soil moisture range with no water stress for cotton. Following the definition of water stress proposed by Luna et al. (2015) [19], soil water content maintained at 45–55% of FC and 60–70% of FC was defined as moderate water stress and mild water stress, respectively, while 90–100% of FC was defined as full irrigation.

The experimental design was a single-factor experiment with three replicates. The study included four different drip irrigation treatments: W1 (3750 m^3^/ha): Soil water content maintained at 45–55% of FC, moderate water stress. W2 (4500 m^3^/ha): Soil water content maintained at 60–70% of FC, mild water stress. W3 (5250 m^3^/ha): Soil water content maintained at 75–85% of FC, suitable moisture (no stress, well-watered control). W4 (6000 m^3^/ha): Soil water content maintained at 90–100% of FC, full irrigation.

Drip irrigation started in mid-June and ended in mid-August, with irrigation performed eight times during the growing season. Detailed irrigation design for each plot is presented in Table 1. The experimental site in Alar, Xinjiang, has an arid continental climate with mean annual precipitation of only 40.1–82.5 mm and mean annual evaporation of 1976.6–2558.9 mm. Therefore, rainfall during the cotton growing season is negligible, and irrigation followed a fixed weekly schedule without weather-based adjustments. The cotton variety Xinluzhong-38 was sown from April 12 to April 16 and harvested during late September.

### 2.3. Soil Samples Collected

Soil samples were collected between the jujube and cotton rows during the cotton seed, bud, boll, and boll-open stages. Three replicates of each soil sample were randomly taken within the orchards (at 0–20 cm depth) and then mixed. The samples were transported in an ice box to the laboratory, where they were sieved through a 2 mm mesh to remove plant debris and soil fauna. Each of the samples was divided into two portions. One portion was air-dried (to standardize moisture conditions and ensure comparability across samples) for the determination of soil chemical properties and enzymatic activities (collected in both 2017 and 2018). The other portion was stored at −20 °C to determine populations of cultivable microbes and soil bacterial community diversity analysis (collected only in 2018). For the microbial analysis, DNA extraction and sequencing were completed in December 2018. The samples were stored continuously at −20 °C from collection until DNA extraction and were thawed only once. This storage duration is common in soil microbial diversity studies, and the absence of freeze–thaw cycles ensures DNA integrity, which was further confirmed by quality control metrics.

### 2.4. Soil Water Content Measurement

Soil samples were collected from the 0–20 cm depth (the main root zone of cotton) at each cotton growth stage (budding, flowering, and bolling stages) for both 2017 and 2018. Three replicate samples were taken randomly from each plot.

Soil water content was determined gravimetrically using the oven-drying method. Fresh soil samples (approximately 20 g) were placed in pre-weighed aluminum boxes, weighed immediately, and then dried in an oven at 105 °C to constant weight. Soil water content (%) was calculated as:

Soil water content (%) = [(Wet soil mass − Dry soil mass)/Dry soil mass] × 100

Soil field capacity (FC) was determined using the standard cutting ring method. Undisturbed soil cores were saturated with water and allowed to drain for 24 h under gravity to remove gravitational water. The remaining soil water content was defined as field capacity.

The percentage of field capacity (% FC) values was used to quantify the water stress levels for each treatment. % FC for each sample was calculated as:

% FC = (Soil water content/FC) × 100

The measured % FC values confirmed that the target soil water content ranges were maintained throughout the growing season: 44–59% FC for W1 (moderate water stress), 64–72% FC for W2 (mild water stress), 74–83% FC for W3 (no stress), and 99–109% FC for W4 (full irrigation) (Table S1). Detailed soil water content data are presented in Supplementary Table S1.

### 2.5. Determination of Soil Chemical Properties and Nutrient Levels

Referring to “Soil Agricultural Chemistry Analysis” [20], OM was deter-mined using the potassium bichromate titrimetric method. Total nitrogen (TN) was estimated using Kjeldahl digestion (KDN-20C, TOP Cloud-agri, Zhenjiang, China). Alkali-hydrolysable nitrogen (AN) was determined using the diffusion method. Available phosphorus (AP) was extracted with 0.5 M NaHCO_3_, and then its content in solution was calculated using the molybdenum antimony coloration determination. Available potassium (AK) was extracted using 1 M CH_3_COONH_4_ and analyzed via flame photometry (FP6420, CNAY, Shanghai, China). Electrical conductivity (TS) was determined using digital conductivity instrument (DDS-11A, REX, Shanghai, China).

### 2.6. Cotton Yield

Cotton yield was determined by hand harvesting of each plot in each treatment on 10 October 2017 and 2018. Then the yield per hectare of cotton was estimated.

### 2.7. Determination of the Activities of Six Soil Enzymes

Activities of urease (UE) (SUE-2-Y), sucrase (SC) (SSC-2-Y), alkaline phosphatase (AKP) (SAKP-2 W), polyphenol oxidase (PPO) (SPPO-2-Y), catalase (CAT) (SCAT-2-Y), and alkaline protease (ALPT) (SALPT-2-Y) were measured using assay kits from Suzhou Keming Biotechnology Co., Ltd. (Suzhou, China), following the manufacturer’s instructions. Colorimetric determinations were performed using a spectrophotometer (UV-5200, Shanghai Precision Instrument Co., Ltd., Shanghai, China). ALPT activity was determined at 680 nm, AKP activity at 660 nm, UE activity at 578 nm, PPO activity at 430 nm, CAT activity at 240 nm, and SC activity at 240 nm. One unit of UE activity was defined as the amount of enzyme that produced 1 μmol NH_3_-N·d^−1^·g^−1^ dry soil. One unit of SC activity was defined as the amount that produced 1 μmol glucose·d^−1^·g^−1^ dry soil. One unit of AKP activity was defined as the amount that produced 1 μmol phenol·d^−1^·g^−1^ dry soil. One unit of PPO activity was defined as the amount that decomposed 1 μmol pyrogallol·h^−1^·g^−1^ dry soil. One unit of CAT activity was defined as the amount that consumed 1 μmol H_2_O_2_·d^−1^·g^−1^ dry soil (substrate-based). One unit of ALPT activity was defined as the amount that produced 1 μmol amino acids·d^−1^·g^−1^ dry soil. Enzyme activities are expressed based on the amount of product produced per unit time, except for CAT, which is based on the amount of substrate (H_2_O_2_) consumed. All units have been uniformly converted to μmol·d^−1^·g^−1^ dry soil. Substrate-free and soil-free controls were included for each sample to eliminate background interference.

### 2.8. Culture and Enumeration of Culturable Soil Bacteria

Soil samples (1 g) were placed into a 250 mL flask containing 9 mL of sterilized distilled water together with glass beads. The flask was sealed with parafilm and shaken at 180 rpm for 30 min at 25 °C to fully disperse soil particles and separate microbial cells from soil. The resulting suspension was designated as the 10^−1^ soil bacterial suspension. An aliquot of 100 µL of this suspension was added to 900 µL of sterile water to obtain a 10^−2^ dilution. Serial dilutions were prepared sequentially up to 10^−5^ for subsequent spread plating.

Bacteria were isolated using the dilution spread-plate method. An aliquot of 100 µL of the appropriate dilution was pipetted onto Petri dishes containing solidified culture medium. The suspension was evenly spread across the surface using a sterilized glass spreader until fully absorbed by the medium. The dilutions selected for bacterial isolation ranged from 10^−4^ to 10^−6^. Plates were incubated in darkness at 37 °C for 1–3 days. Colonies were counted from plates at the 10^−5^ dilution. After inoculation, all Petri dishes were sealed with plastic wrap and incubated upside down. At the end of incubation, colonies were counted, and the average of three replicates was calculated.

The number of culturable bacteria per gram of dry soil was calculated using the following formula:

Number of colony-forming units (CFU) per gram of dry soil = average number of colonies per plate × dilution factor

### 2.9. Determination of Soil Bacterial Diversity

Soil subsamples (50 g each) stored at −20 °C were used for crude total DNA extraction by the SDS high-salt method. The crude DNA was then purified using an Ezup Column Soil DNA Purification Kit (B518263, Sangon Biotech, Shanghai, China) according to the manufacturer’s instructions. The purified DNA was stored at −20 °C for 16S rDNA amplicon sequencing on the IonS5TMXL platform (Novogene, Beijing, China).

### 2.10. Data Analysis and Processing

In order to make the result of information analysis more accurate and reliable, the IonS5TMXL offline data is exported to fast file. The data of each sample is distinguished according to the barcode sequence. Then, the chimeric filter is used to obtain the effective data for subsequent analysis.

Uparse software (version 7.0.1001) [21] was used to cluster all clean reads of all samples. By default, 97% identity was used to cluster the sequences into OTUs (operational taxonomic units), and then the representative sequences of OTUs were annotated.

The OTUs sequences were annotated and analyzed by the mothur method and SSUrRNA [22] database of silva132 [23] (threshold value was 0.8 ~ 1). The taxonomic information was obtained and the community composition of each sample was counted at genus level. According to the species annotation and abundance information of all samples at the genus level, the 35 genera with the highest abundance were selected. According to the abundance information in each sample, the species and samples were clustered, and the heat map was drawn to find out which species were clustered more or less abundant in which samples.

QIIME software (version 1.9.1) was used to calculate the Chao1 and Shannon indexes. To assess the differences in species richness and diversity of microbial communities in each sample.

Beta diversity was assessed using principal coordinate analysis (PCoA) based on Bray–Curtis distances. Permutational multivariate analysis of variance (PERMANOVA, Adonis) with 999 permutations was performed to test the significance of treatment effects on microbial community composition. Using R software (version 2.15.3), Wilcoxon rank-sum test of Agricola package were used to analyze the difference in beta diversity index between groups.

VPA (variance partitioning canonical response analysis) [24], RDA (x, y, z) in vegan package was used to analyze the effects of main environmental factors (y) and co-environmental factors (z) on species distribution (x), and the contribution degree of each environmental factor causing the difference in microbial community distribution could be obtained.

Spearman rank correlation was used to study the relationship between environmental factors and microbial species richness (alpha diversity), and the relationship between environmental factors and species was studied. The correlation and significant *p*-values between environmental factors and species were obtained. In the Spearman correlation analysis, we first calculated the Spearman correlation coefficients between species and environmental factors and tested their significance. Then, the heatmap function in the heatmap package was used for visualization.

### 2.11. Statistical Analyses

All data are reported as means ± standard deviations. Statistical procedures were conducted in the software package SPSS (version 24.0). One-way ANOVA and Duncan’s test were conducted to determine significant differences among the four treatments considering *p* < 0.05 as the threshold for significance.

## 3. Results

### 3.1. Effect of Water Stress on Soil Nutrient and Crop Yield in an Intercropping Cotton Field

Significant differences in soil nutrients and cotton yield were observed between different irrigation rates (Table 2, *p* < 0.05). Under different irrigation rates, W2 and W3 treatments generally enhanced soil nutrient levels compared to W1 and W4. AP was significantly higher under W3 (135.88 ± 7.2 mg/kg) than under all other treatments (*p* < 0.05). AN was highest under W2 (112.57 ± 4.3 mg/kg), significantly exceeding that under W3 (99.05 ± 2.4 mg/kg) (*p* < 0.05). AK under W2 and W3 was comparable to W1 and notably higher than under W4. TS was lower under W2 and W3, indicating less salinity stress. TN and OM did not differ significantly among treatments (*p* > 0.05).

Cotton yield in intercropping system was also significantly influenced by irrigation rate treatment (*p* < 0.05). W3 treatment resulted in the highest cotton yield (1973.49 kg/ha), representing a significant increase of 52.51% compared to W1 (1293.98 kg/ha, the lowest value), but there was no significant difference from W2 (1780.06 kg/ha).

Overall, soil nutrient levels and cotton yield increased under W2 and W3 treatments.

### 3.2. Effect of Water Stress on Soil Enzyme Activity in an Intercropping Cotton Field

Enzymatic activities were presented in Table S2. During the cotton growing period, under W2 and W3 treatments, CAT and UE activities showed an increasing trend, while AKP and ALPT activities decreased. PPO and SC activities initially increased and then decreased, reaching their peak at the seedling and budding stages, respectively. Two-way ANOVA showed that year had significant or extremely significant effects on all indicators (*p* < 0.01 or *p* < 0.001). The irrigation rate significantly affected CAT (*p* < 0.01), AKP (*p* < 0.001), UE (*p* < 0.01), and SC (*p* < 0.001) activities, but had no significant effect on ALPT and PPO (*p* > 0.05) activities. Only SC activities exhibited a significant irrigation rate × year interaction (*p* < 0.01), while the interactions for the other indicators were not significant (*p* > 0.05) (Table 3). Due to the significant irrigation rate × year interaction effect on SC activities, further simple effects analysis was conducted. The results showed that in 2018, the irrigation rate significantly affected SC activities, whereas no significant change was observed in 2017 (Table S3).

### 3.3. Taxon Distribution and Impact of Water Stress on Soil Bacterial Community Composition

An average of 83,449 bacterial reads were obtained from each sample analyzed on the IonS5TMXL platform using single-end sequencing. After quality control, an average of 77,940 effective reads per sample was obtained (Table S4), and 8450 operational taxonomic units (OTUs) were clustered at 97% sequence identity. Of these OTUs, 8447 (99.96%) were annotated to the domain level using the SILVA132 database. Using the same database, 91.28% of OTUs were annotated to the phylum level, 79.46% to the class level, 65.17% to the order level, 55.34% to the family level, 36.01% to the genus level (3043 OTUs), and 9.29% to the species level.

**Figure 1:**
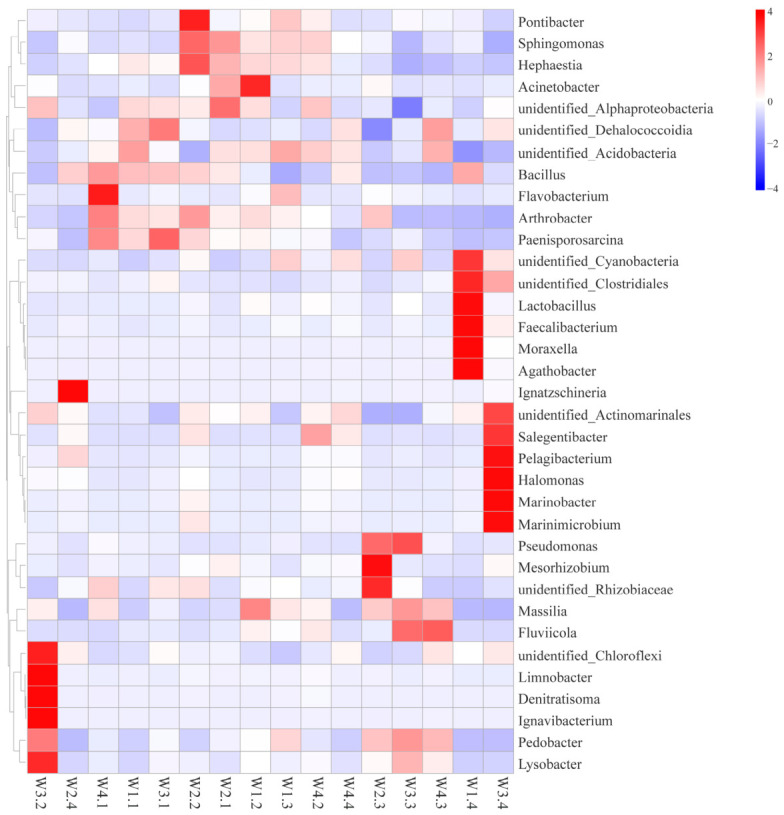
The soil bacteria community composition from different water treatment. The number behind the moisture indicates the growth period: 1 (Seedling stage), 2 (Budding stage), 3 (Flowering stage), 4 (Bolling stage).

Water stress significantly affected the bacterial community composition (Figure 1). Based on the annotation and abundance data at the genus level, the top 35 genera were selected and clustered for further analysis. In response to increasing water stress gradients, 20 bacterial genera exhibited higher abundance in soil samples, including *Pontibacter*, *Ignatzschineria*, *Limnobacter*, *Pseudomonas*, *Mesorhizobium*, *Denitratisoma*, *Ignavi-bacterium*, *Lysobacter*, *Pelagibacterium*, *Halomonas*, *Marinimicrobium*, and *Marinobacter*. Genera *Pseudomonas* significantly increased in W2 and W3 treatments but decreased in W1 and W4 treatments. These 20 genera might thus be responsive to irrigation rate.

In addition, culturable bacterial counts were determined using the dilution spread-plate method. The results showed that under water stress conditions, the highest soil bacterial counts were observed in the W2 and W3 treatments (Figure S1), indicating that moderate water stress promoted soil bacterial proliferation during these specific growth stages. These culture-dependent results are consistent with the sequencing data, which also revealed higher abundance of several bacterial genera under W2 and W3 conditions.

### 3.4. Effects of Water Stress on the α-Diversity of Cotton Rhizosphere Soil Bacterial Communities

**Figure 2:**
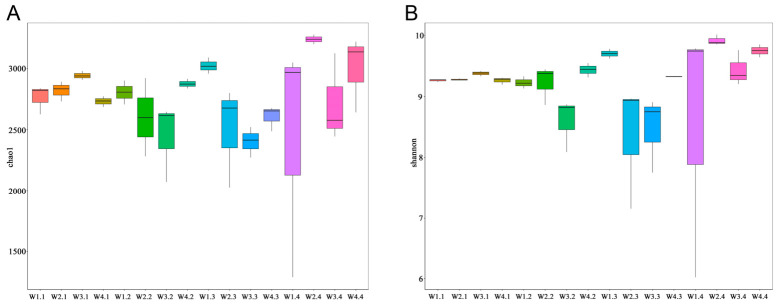
Soil bacterial alpha diversity in an intercropping cotton field. The number behind the moisture indicates the growth period: 1 (Seedling stage), 2 (Budding stage), 3 (Flowering stage), 4 (Bolling stage). (**A**) Chao1 index of cotton rhizosphere soil; (**B**) Shannon index of cotton rhizosphere soil.

Significant differences in species alpha-diversity among groups were observed based on the Wilcoxon rank-sum test (Figure 2). Irrigation rate significantly affected microbial alpha-diversity in the cotton rhizosphere during the flowering and bolling stages. The Chao1 (Figure 2A) and Shannon (Figure 2B) indices were significantly higher during the bolling stage under W2 treatment.

### 3.5. Effects of Water Stress on the β-Diversity of Cotton Rhizosphere Soil Bacterial Communities

**Figure 3:**
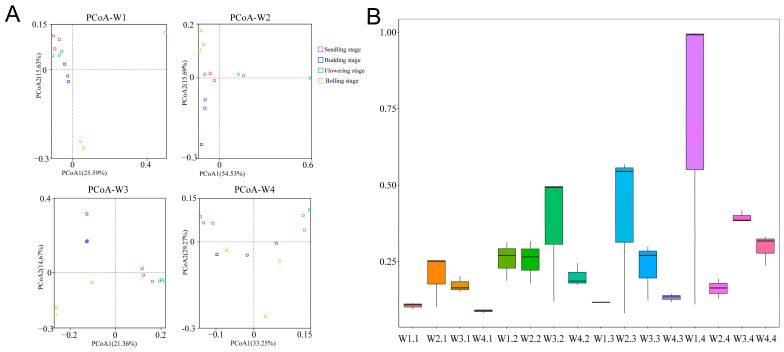
Influence of water stress on the beta-diversity of cotton rhizosphere soil bacterial communities. (**A**) Loadings for Principal Coordinate Analysis (PCoA) of microbial diversity by soil water stress in intercropping orchard. (**B**) Box Plot for analysis of difference between beta-diversity groups. The number behind the moisture indicates the growth period: 1 (Seedling stage), 2 (Budding stage), 3 (Flowering stage), 4 (Bolling stage).

We compared the diversity of microbial community structure across different irrigation treatments in the intercropping system using principal coordinate analysis (PCoA; Figure 3A). The result indicated that irrigation rate significantly affected microbial community composition at the seeding, budding, flowering, and bolling stages (Figure 3A). PERMANOVA revealed that treatment combination explained 57.5% of the total variation (R^2^ = 0.575) and the effect was highly significant (*p* = 0.001) (Table S5). Wilcoxon rank-sum test was used to analyze differences in species beta-diversity revealed significant differences in microbial community structure between W2 and W3, and other treatments (Figure 3B).

### 3.6. Effects of Soil Enzyme Activities and Nutrient Levels on the Structure and Diversity of Soil Bacterial Communities

**Figure 4:**
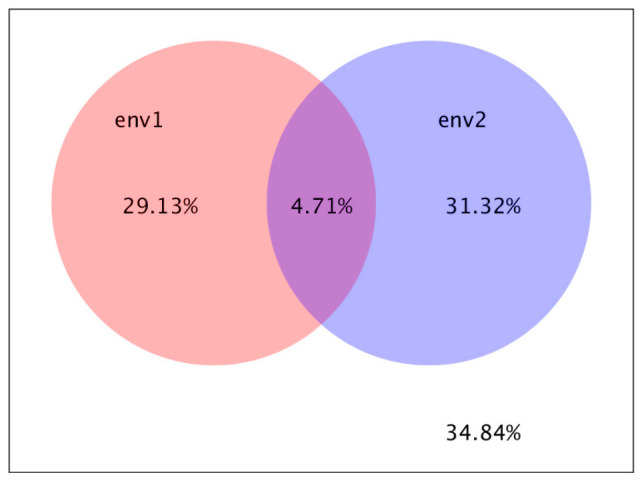
The variance partitioning canonical correspondence analysis (VPA) between soil bacterial communities and environmental factors. env1 (TN, AN, AP, AK, OM, TS), env2 (PPO, CAT, UE, SC, ALPT, APK).

Correlations between soil enzymatic activity, soil nutrient levels, and other environmental factors and microbial community structure were obtained using variance partitioning canonical correspondence analysis (VPA) (Figure 4). The environmental factors (env) were divided into two categories and VPA was conducted: evn1 (soil nutrients: TN, AN, AP, AK, OM, TS) and env2 (soil enzymes: PPO, CAT, UE, SC, ALPT, AKP) accounted for 29.13% and 31.32% of the differences observed in bacterial community structure, respectively. The coupling effect of the two categories had a 4.71% impact on the microbial community and the remaining 34.84% could not be explained by the environmental factors considered.

**Figure 5:**
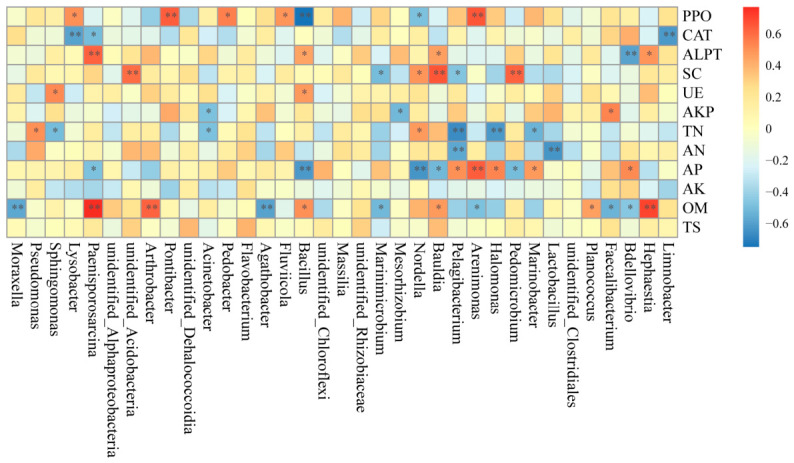
The spearman Correlation analysis between soil bacterial communities and environmental factors. TS, soil conductivity; AN, alkali-hydrolysable nitrogen; TN, total nitrogen; AP, available phosphorus; AK, available potassium; OM, Organic matter; ALPT: alcalase protease; AKP: alkaline phosphatase; UE: urease; PPO: Polyphenol oxidase; SC: sucrase; CAT: catalase. The vertical direction is the environmental factor information, and the horizontal direction is the species information. The corresponding value of the middle heat map is Spearman’s correlation coefficient r, which is between −1 and 1. r < 0 is negative correlation, r > 0 is positive correlation, and the mark * indicates significant, the *p*-value of sex test was <0.05, ** indicates significant, the *p*-value of sex test was <0.01.

Correlations between environmental factors and OTUs were evaluated through Spearman’s correlation analysis. Among the 50 bacterial genera identified in OTUs, the 35 most abundant genera had significant effects on soil enzymatic activity and soil nutrient levels. Among these, 20 genera significantly affected soil enzymatic activity, and 23 genera significantly affected soil nutrient levels (Spearman’s r > 0.46 or < −0.46, *p* ≤ 0.05) (Figure 5). Soil PPO activity and AP were significantly correlated to genera Pontibacter (r = 0.6361, *p* = 0.0025) and Arenimonas (r = 0.6391, *p* = 0.0024) in the W2 treatment, respectively. Soil AP was significantly correlated to genus Halomonas (r = 0.5338, *p* = 0.0153) in the W3 treatment. Soil TN was significantly correlated to genus *Pseudomonas* (r = 0.5069, *p* = 0.0225) in the W2 and W3 treatments.

## 4. Discussion

### 4.1. Response of Cotton Yield to Water Stress

Cotton has a moderate water demand. Although cotton is considered drought-tolerant and can survive in rain-free environments, irrigation is still required to achieve acceptable yields, especially under intercropping conditions in arid regions. This study confirmed that water stress significantly affects cotton yield in a jujube/cotton intercropping system. Our results showed that cotton yield was significantly higher under the W3 treatment compared to the water-stress treatments (W1 and W2), but no significant difference was observed between W3 and W2 (Table 2). This finding suggests that a moderate reduction in irrigation (from 5250 to 4500 m^3^/ha) may achieve comparable yields while saving approximately 14% of irrigation water.

Our results are consistent with previous studies. Moderate water stress has been reported to positively affect root growth and yield, with more drought-tolerant varieties showing higher yields under appropriate water stress [25]. This phenomenon may be related to the reduction in cotton plant height [26], as plant height significantly influences the number of fruiting branches, which in turn determines boll number and final yield. In addition, leaf photosynthetic capacity regulates boll weight. As water stress intensity increases, the relative water content of main stem leaves and sympodial leaves gradually decreases, while the number of cotton seeds per plant is significantly reduced, both of which are key factors determining cotton yield [27].

Young jujube orchards typically yield less due to smaller trees, but they benefit from abundant sunlight. This resource is maximized by planting cotton, which also improves income. Under this system, cotton yield remains stable even after five years of continuous cropping. However, in water-scarce regions, intercropping systems generally require more water than monocultures. Nevertheless, our study demonstrates that using mild water stress W2 not only maintains cotton yield comparable to conventional field irrigation W3 but also significantly reduces water consumption. This finding has important implications for irrigation management in arid regions, where water resources are increasingly limited.

### 4.2. Response of Soil Nutrient Levels to Water Stress

An agroforestry intercropping system can enhance the farmland microclimate and improve the efficiency of land, light, heat, water, and fertilizer utilization [8]. Our results support this conclusion, as the jujube/cotton intercropping system maintained stable soil nutrient levels and cotton yield even under reduced irrigation conditions.

Intercropping significantly affects root biomass by increasing plant diversity, and the environmental changes caused by root growth influence soil nutrient levels. Under different water stress treatments, a nonlinear relationship was observed between soil nutrient levels and water supply, with significant increases in soil AP and AK in W2 and W3 treatments (Table 2). This suggests that irrigation affects the dissolution and migration of nutrients in the soil [28], and excess water may accelerate nutrient leaching and loss.

In arid regions with light-textured soils, frequent and high irrigation volumes can push mobile nutrients below the root zone (0–20 cm), making them unavailable to cotton. This is consistent with previous findings that excessive irrigation reduces nitrogen and potassium retention in the root zone [29], which aligns with our observation that AK and AP decreased under the W4 treatment (Table 2). On the other hand, under W1, limited soil moisture restricts nutrient diffusion in the soil solution, reducing the contact between roots and available nutrients. In addition, water stress suppresses microbial activity, leading to reduced nutrient mineralization [30]. This may help explain why AN and AP were lowest under W1 (Table 2).

In Xinjiang, salt stress is a common limiting factor affecting cotton growth [31]. Interestingly, soil EC was lower under W2 and W3, suggesting that moderate irrigation (60–85% FC) helps maintain a favorable salt balance by avoiding both salt accumulation. The higher EC under W1 (Table 2) is consistent with previous findings that insufficient irrigation leads to salt accumulation in the surface soil layer due to reduced leaching [32]. Our results highlight the trade-off between water saving and salinity control, providing an effective irrigation reference range (4500–5250 m^3^/ha) for arid intercropping systems.

### 4.3. Response of Soil Enzymes to Water Stress

Soil enzymatic activities are closely linked to nutrient conversion, influencing soil OM decomposition and the release of mineral elements in the soil [33]. Soil enzyme activities are particularly high during the crop growing period [12]. Our data showed that soil enzymes, such as ALPT, PPO, and UE, were most active during the flowering and bolling stages of cotton (Table 3). This may be due to the root and leaf litter of jujube, which could stimulate higher enzyme activities. Our results indicated that intercropping increased the activities of certain soil enzymes by enhancing plant species diversity, a key factor in ecosystem productivity.

Proper irrigation significantly enhances cotton plant growth [34]. However, beyond a certain threshold, increased irrigation negatively affects cotton growth. In this study, soil PPO and SC activities significantly increased under W2 and W3 treatments (Table 3). The higher enzyme activities under W2 may be explained by mild water stress stimulating root exudation and microbial activity, which is consistent with previous findings in other crop species. Under mild stress, plants allocate more carbon to roots, increasing root exudates that serve as substrates for enzyme-producing microorganisms [35]. In contrast, severe water stress suppresses microbial metabolism and reduces enzyme synthesis [36], while excess irrigation may lead to oxygen limitation and reduced aerobic microbial activity [37].

Soil PPO typically forms humus-like substances through biochemical reactions, and its activity reflects the soil’s remediation capacity. Soil SC produces glucose, which can be directly absorbed by plants. Our results suggest that a moderate reduction in water supply (W2) in the intercropping system helps to enhance soil enzyme activity, while both severe deficit (W1) and excess irrigation (W4) are detrimental (Table 3). Increased enzyme activity further demonstrates that appropriate irrigation rates promote the transformation of soil humus-like substances and accelerate glucose production.

### 4.4. Response of Environment to Soil Bacterial Community Structure

The intercropping system enhances the complexity of ecological functions through the interaction of multiple crops, significantly influences the community composition and diversity of soil microorganisms. The present study demonstrated that the richness and diversity of the microbial community structure significantly increased in W2 and W3 treatments, while they decreased in W1 and W4 treatments (Figure 2 and Figure 3). This may be attributed to the reduced rate of soil microbial metabolism under drought conditions, which decreased microbial activity and abundance [38]. Additionally, excessive irrigation does not improve soil microbial activity or soil health [39]. Furthermore, soil moisture is considered one of the primary factors influencing the abundance and structure of soil microorganisms [40], as it regulates osmotic potential and microbial cell metabolism, thereby affecting microbial activity and metabolism [41].

Interestingly, the bacterial genus *Pseudomonas* was significantly more abundant under the W2 and W3 than in the other treatments (Figure 1). *Pseudomonas* belongs to the PGPB (Plant Growth Promoting *Rhizobacteria*) genera, which improves salt tolerance in crops, promotes plant growth, and inhibits bacterial infection [41], and can therefore be used as a control agent for biological diseases and for enhancing plant resistance [42]. As cotton planting years increase, soil diseases gradually appear. Thus, the W2 and W3 treatments showed higher soil microbial richness and strong resistance when the period of cotton intercropping was longer. However, soil microbial community is also affected by soil physical and chemical properties and plant species [43,44]. *Pontibacter*, *Arenimonas*, *Halomonas*, and *Pseudomonas* were the most abundant genera in W2 and W3 treatments (Figure 5) and were significantly positively correlated with soil AP, TN, and PPO. These correlations suggest that, under specific water stress conditions, certain stress-tolerant microorganisms become more prevalent. Whether these microorganisms actively improve plant stress resistance or simply respond to favorable soil conditions cannot be determined from the current correlational data [30]. The observed associations may, at least in part, reflect microbial responses to altered soil environments rather than direct causal effects on crop yield.

These results showed that under the intercropping system, water greatly affects soil water content, which in turn influences microbial diversity and enzymatic activities. However, it is the microbial community structure and activity that largely determine soil enzymatic activity, rather than the reverse. Given the correlational nature of our data, we cannot conclude that specific bacterial genera directly influence enzyme activity or nutrient content. Nevertheless, the observed associations suggest that moderate irrigation (W2) promotes favorable soil conditions, including higher enzyme activities and nutrient availability, which are associated with increased cotton yield.

## 5. Conclusions

**Figure 6:**
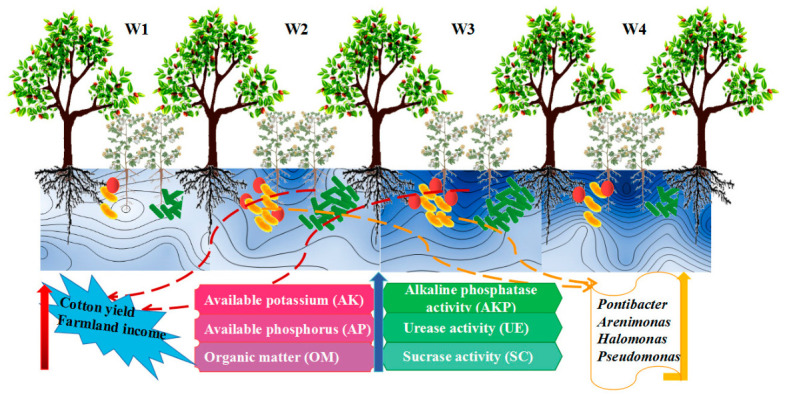
Schematic representation of the water-stress-driven trade-offs among soil physicochemical properties, enzyme activities, and microbial diversity across cotton growth stages under intercropping. Red boxes indicate increased enzyme activity types; green boxes indicate increased soil nutrient types; yellow dashed lines represent increased soil bacterial types under the corresponding treatments; red dashed lines represent increased cotton yield and agricultural income under the corresponding treatments.

Our results indicated that, in a cotton intercropping system, water stress effects on soil nutrient levels, enzymatic activity, and soil bacterial abundance varied significantly across different cotton growth stages. The W2 and W3 treatments were associated with significantly higher cotton yield, higher soil AP and AK, higher AKP and UE activities, and distinct microbial community structures, including a higher relative abundance of *Pseudomonas* (Figure 6). However, the observed associations may reflect microbial responses to altered soil conditions rather than direct effects on yield or nutrient cycling. Considering both cotton yield and water use efficiency, an irrigation volume between 4500 and 5250 m^3^/ha is recommended for the jujube/cotton intercropping system in arid regions of southern Xinjiang. These findings have implications for resource management in intercropping systems and provide a basis for implementing water-saving measures.

## Figures and Tables

**Table 1:** Irrigation design for each treatment plot.

Treatment	Total Irrigation (m^3^/ha)	Plot Area (ha)	Irrigation Frequency	First Irrigation (m^3^)	Each of 2nd–8th Irrigations (m^3^)
W1	3750	0.029	8	15	13.38
W2	4500	0.029	8	15	16.49
W3	5250	0.0319	8	15	21.77
W4	6000	0.0319	8	15	25.19

Note: The first irrigation volume was kept constant for seedling establishment.

**Table 2:** Effect of water stress on the change in soil fertility and yield in an intercropping cotton field.

Parameter Name		W1	W2	W3	W4
TS (μs/cm)		270.42 ± 3.6 a	249.58 ± 2.0 ab	225.67 ± 15.3 b	238.92 ± 12.7 b
AN (mg/kg)		103.27 ± 4.6 ab	112.57 ± 4.3 a	99.05 ± 2.4 b	106.56 ± 4.7 ab
TN (mg/kg)		975.30 ± 95.0 a	879.70 ± 105.3 a	917.00 ± 57.3 a	819.00 ± 37.0 a
AK (mg/kg)		170.28 ± 19.8 a	149.65 ± 10.3 a	153.35 ± 9.5 a	121.62 ± 2.5 b
AP (mg/kg)		96.66 ± 6.9 b	98.18 ± 13.1 b	135.88 ± 7.2 a	106.27 ± 2.4 b
OM (mg/kg)		21,019.4 ± 937.8 a	21,513.10 ± 1377.9 a	20,510.30 ± 2110.6 a	19,556.5 ± 1196.8 a
Lint Yield (kg/ha)	2017	1225.04 ± 91.1 b	1758.52 ± 107.6 a	1937.99 ± 299.4 a	1414.07 ± 72.2 b
	2018	1362.92 ± 189.3 c	1801.60 ± 42.8 ab	2008.98 ± 270.1 a	1460.70 ± 42.5 bc
	mean	1293.98 ± 152.8 b	1780.05 ± 77.0 a	1973.49 ± 258.0 a	1437.38 ± 58.8 b

Note: TS, electrical conductivitys; AN, alkali-hydroly sablenitrogen; TN, total nitrogen; AP, available phosphorus; AK, available potassium; OM, Organic matter. Data are presented as means of three replicates ± SD, *n* = 3. Means with different letters have significant differences in same stage (*p* < 0.05; Duncan’s test).

**Table 3:** Changes in soil enzyme activity.

Parameter Name	W1	W2	W3	W4	Source of Variation		
					Irrigation Rate	Year	Irrigation Rate × Year
ALPT (μmol·d^−1^·g^−1^)	326.96 ± 93.0 a	326.30 ± 128.6 a	297.62 ± 99.8 a	332.26 ± 108.7 a	1.48	217.90 ***	1.25
PPO (μmol·d^−1^·g^−1^)	139.05 ± 50.8 ab	126.46 ± 33.6 b	140.62 ± 58.0 a	134.23 ± 49.7 ab	0.43	78.72 **	5.19
CAT (μmol·d^−1^·g^−1^)	34.51 ± 13.4 bc	38.27 ± 15.9 a	36.32 ± 16.3 ab	32.66 ± 13.5 c	6.37 **	785.17 ***	2.17
AKP (μmol·d^−1^·g^−1^)	9.80 ± 1.4 a	9.75 ± 1.7 a	8.61 ± 1.5 b	7.60 ± 1.3 c	14.50 ***	73.46 ***	0.87
UE (μmol·d^−1^·g^−1^)	16.39 ± 7.9 a	16.59 ± 8.3 a	13.20 ± 7.8 b	14.86 ± 6.2 c	5.86 **	435.58 ***	1.38
SC (μmol·d^−1^·g^−1^)	326.96 ± 93.0 a	326.30 ± 128.6 a	297.62 ± 99.8 a	332.26 ± 108.7 a	10.81 ***	323.13 ***	8.25 **

Note: ALPT: alcalase protease, AKP: alkaline phosphatase, UE: urease, PPO: Polyphenol oxidase, SC: sucrase, CAT: catalase. Data are presented as means of all growth stages across two years (2017, 2018) ± SD, *n* = 24. Means with different letters have significant differences in same stage (*p* < 0.05; Duncan’s test). Values in the source of variation column represent F-statistics from two-way ANOVA with Duncan’s test. Significance levels are denoted as follows: **, *p* < 0.01; ***, *p* < 0.001.

## Data Availability

The original contributions presented in this study are included in the article. Further inquiries can be directed to the corresponding authors.

## Supplementary Materials

The following supporting information can be downloaded at: https://www.mdpi.com/article/10.3390/microorganisms14071413/s1, Figure S1. Soil culturable bacterial abundance at different growth stages under varying water stress conditions. Data are shown as mean ± SD (*n* = 9), Means with different letters have significant differences in same stage (*p* < 0.05; Duncan’s test). Table S1. Soil water content and percentage of field capacity at different cotton growth stages under different irrigation treatments; Table S2. Changes in soil enzyme activity of stage; Table S3. Effects of irrigation rate on SC activity (μmol·d^−1^·g^−1^) separated by year; Table S4. Overview of sequencing data; Table S5. PERMANOVA results comparing microbial community composition among treatments based on Bray–Curtis distances.
