# Supplementary figures and images for: Effects of Irrigation Rate on Soil Bacterial Diversity and Lint Yield in a Jujube/Cotton Intercropping System
Source: Microorganisms. 2026 Jun 27;14(7):1413. doi: 10.3390/microorganisms14071413 (PMC13413598; doi:10.3390/microorganisms14071413)

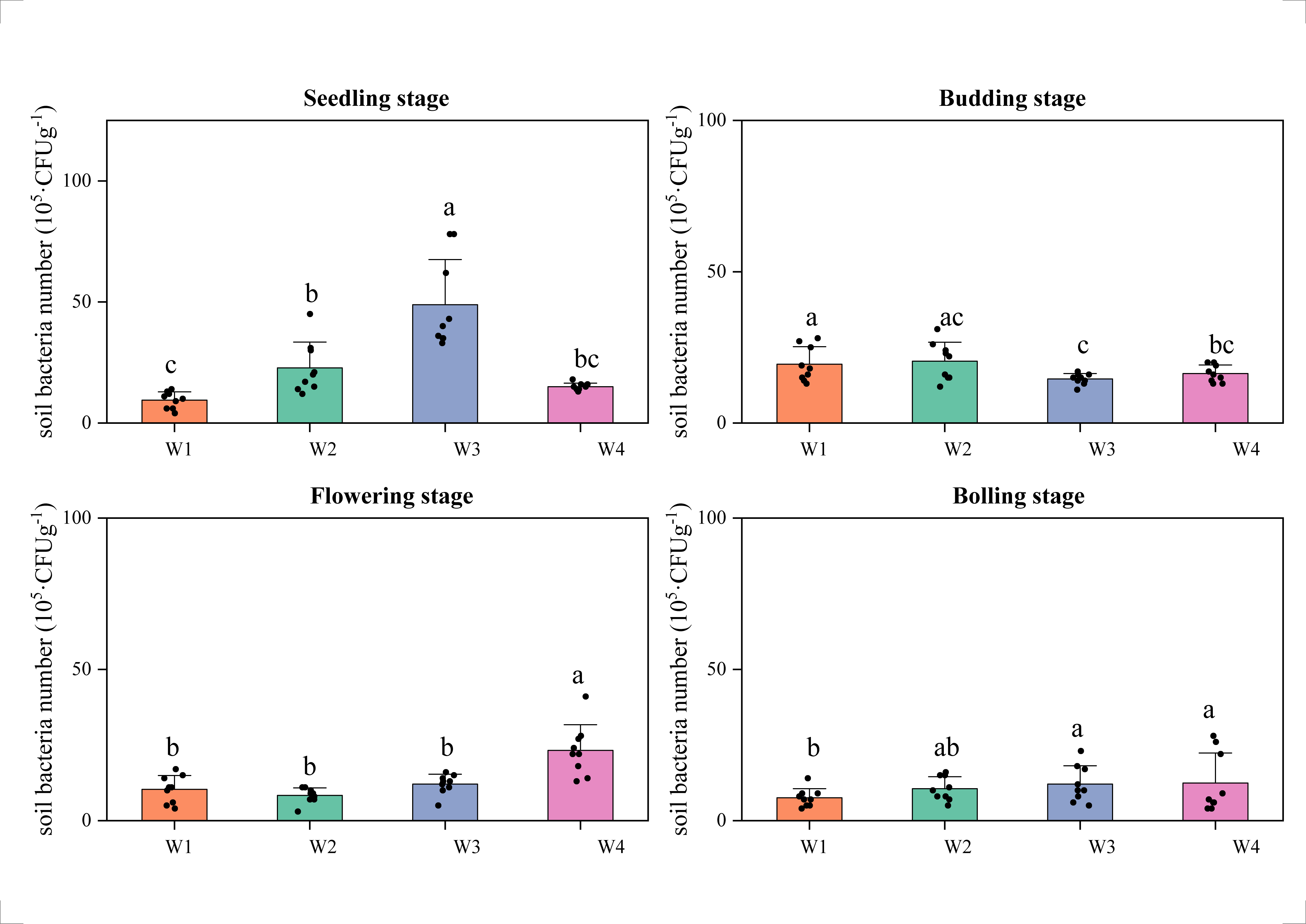

Supplement: Supplementary file 1 [file microorganisms-14-01413-s001.zip › Figure S1.tif]
